# LAG-3 Inhibitors: Novel Immune Checkpoint Inhibitors Changing the Landscape of Immunotherapy

**DOI:** 10.3390/biomedicines11071878

**Published:** 2023-07-01

**Authors:** Rebecca Ibrahim, Khalil Saleh, Claude Chahine, Rita Khoury, Nadine Khalife, Axel Le Cesne

**Affiliations:** 1 International Department, Gustave Roussy Cancer Campus, 94800 Villejuif, France; rebecca.ibrahim@lau.edu (R.I.); claude.chahine@gustaveroussy.fr (C.C.); rita.khoury011@gmail.com (R.K.); axel.lecesne@gustaveroussy.fr (A.L.C.); 2 Department of head and neck Oncology, Gustave Roussy Cancer Campus, 94800 Villejuif, France; khalife.nadine@live.com

**Keywords:** immune checkpoint, immunotherapy, lymphocyte activation gene-3 (LAG-3)

## Abstract

One of the most important steps forward in the management of cancer was the discovery of immunotherapy. It has become an essential pillar in the treatment paradigm of cancer patients. Unfortunately, despite the various options presented with immune checkpoint inhibitors (ICIs), the benefit is still limited to select patients and the vast majority of these patients gain either minimal benefit or eventually progress, leaving an unmet need for the development of novel therapeutic agents and strategies. Lymphocyte activation gene-3 (LAG-3), an immune checkpoint receptor protein, is a molecule found on the surface of activated T-cells. It plays a major role in negatively regulating T-cell function thereby providing tumors with an immune escape in the tumor microenvironment (TME). Given its importance in regulating the immune system, LAG-3 has been considered as a promising target in oncology and precision medicine. To date, two LAG-3-directed agents (eftilagimod alpha and relatlimab) have been approved in combination with programmed death-1 (PD-1) inhibitors in the setting of advanced solid tumors. In this review, we discuss the structure of LAG-3, its mechanism of action, and its interaction with its ligands. We also shed light on the emerging treatments targeting LAG-3 for the treatment of solid tumors.

## 1. Introduction

The discovery of immune checkpoint inhibitors (ICIs) has considerably changed the landscape of cancer treatment in the last few decades by allowing a deeper understanding of the continuously evolving entity of the tumor microenvironment (TME) [1]. It is believed that the interaction between the various components of the TME including immune checkpoints contributes to tumor growth, progression, metastasis, and eventually resistance to various therapies [2,3]. Since 2011, cytotoxic T lymphocyte-associated protein 4 (CTLA-4), programmed cell death protein 1 (PD-1), and programmed death-ligand 1 (PD-L1) inhibitors have been granted approvals by the Food and Drug Administration (FDA) for the treatment of several solid malignancies [4]. In order to overcome resistance to these agents, alternative immune checkpoints in the TME are under investigation, such as lymphocyte activation gene-3 (LAG-3), T-cell immunoglobulin and mucin domain 3 (TIM-3), V-domain immunoglobulin-containing suppressor of T-cell activation (VIST A), and human endogenous retrovirus-H long terminal repeat-associating protein 2 (HHLA2) [5,6,7,8,9].

**Figure 1:**
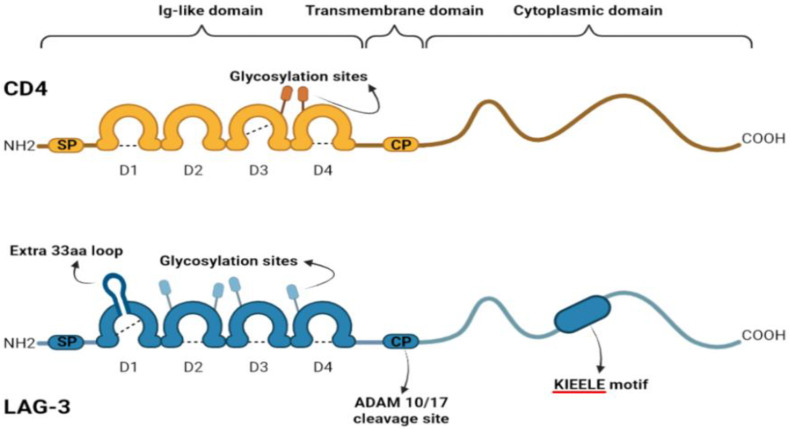
Similarities of CD4 and LAG3.

**Figure 2:**
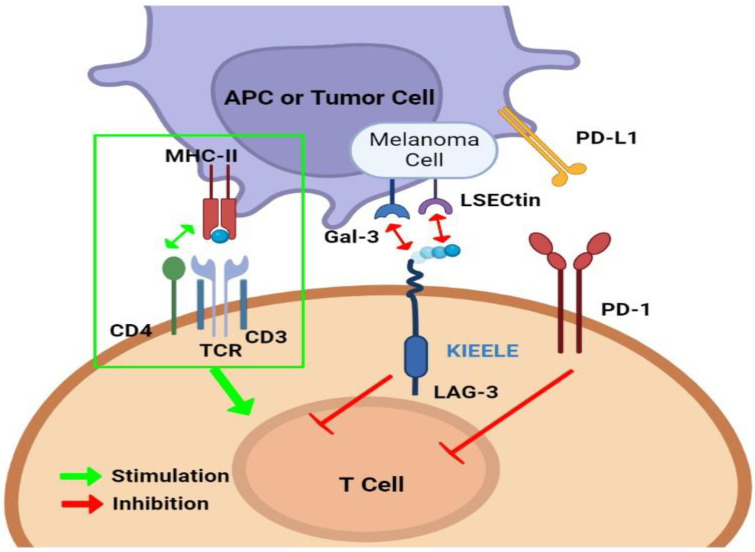
Immune checkpoints and tumor microenvironment.

LAG-3 (CD223) has a regulatory role comparable to PD-L1 and CTLA-4 that consists in the inhibition of immune function, cell proliferation, homeostasis, and cytokine secretion [10,11]. It was first described in 1990 by Triebel et al. as a 498 amino acid transmembrane protein with four extracellular immunoglobulin-like domains and encoded by the *LAG3* gene [12]. The gene is adjacent to the CD4 gene (chromosome 12 in humans). LAG-3 and CD4 share about 20% amino acid sequence homology. Figure 1 shows the similarity of CD4 and LAG-3. This translated into comparable extracellular folding patterns giving LAG-3 the ability to bind to major histocompatibility complex (MHC) class II on antigen-presenting cells (APCs), but with greater affinity than CD4 leading to the inhibition of cellular proliferation and T-cell activation [13,14]. In fact, LAG-3: MHC class II interaction will prohibit the binding of that same MHC molecule to T-cell receptor (TCR) and CD4; therefore, putting a stop to TCR signaling (Figure 2) [15,16]. LAG-3 has two other major ligands in the TME: Galectin-3, a galactoside-binding soluble lectin, which was shown to be a regulator of antigen-specific T-cell activation, and liver sinusoidal endothelial cell lectin (LSECtin) that has a significant inhibitory impact on antitumor response [17,18]. LAG-3 is also associated with the down-modulation of CD3/TCR complex expression. Moreover, LAG-3 transmits inhibitory signals via the KIEELE motif in its cytoplasmic tail further impairing T-cell proliferation [19]. The KIEELE motif (particularly the lysine residue of this motif) has been reported to be indispensable for LAG-3 mediated inhibition of TCR signaling, both in vivo and in vitro; however, the intracellular binding molecules of the KIEELE motif are still not described in the literature [10,20,21]. Taken together, LAG-3-directed therapies constitute a new member of the therapeutic arsenal of cancer patients. Relatlimab, an anti-LAG-3 monoclonal antibody, and eftilagimod alpha (IMP321), a soluble LAG-3 fusion protein are approved by the FDA in combination with PD-1 inhibitors. In the review, we discuss the expression of LAG-3 on immune cells, tumor cells, interaction with other immune checkpoints as well as the LAG-3-directed therapies.

This figure illustrates similarities between CD4 and LAG-3. Each one is composed of three domains: an extracellular region with four domains, one transmembrane domain, and a cytoplasmic domain.

Figure 2 illustrates the interaction of immune checkpoints and tumor microenvironment.

Antigen-presenting cells present tumor antigens to T-cells inducing their activation. The first signal for T-cell activation is mediated by the major histocompatibility complex and TCR signaling pathway. Immune checkpoints inhibit T-cell activation in the tumor microenvironment. LAG-3 immune checkpoint is composed of an extracellular domain with four domains, transmembrane-cytoplasmic domain, and cytoplasmic region. The ligands of LAG-3 in the tumor microenvironment are MHC II, Galectin-3, and LSECtin.

## 2. LAG-3 Structure and Regulation

As previously mentioned, LAG-3 is a type I transmembrane protein. The extracellular region of LAG-3 consisted of four domains sharing approximately 20% of homology with CD4 as previously mentioned. However, the intracellular regions of CD4 and LAG-3 do not share noticeable similarities. In fact, the cysteine motif, which is required for the association with lymphocyte-specific protein tyrosine kinase, and the palmitoylation sites present in CD4 are absent in LAG-3 [22,23]. The biosynthesis of LAG-3 undergoes multiple modifications. One of these steps is the cleavage of LAG-3 at the connecting peptide by the metalloproteinases ADAM10 and ADAM17 leading to the release of the extracellular region of LAG-3 in a soluble form through two distinct mechanisms. ADAM10 induces constitutive LAG-3 cleavage, while ADAM17 mediates LAG-3 cleavage induced by TCR signaling [24,25]. The expression of LAG-3 is mediated by T-cell activation and upregulated by cytokines such as IL-2 and IL-12 [26,27]. LAG-3 expression inversely correlates with IL-4 production [28]. The epigenetic regulation of LAG-3 transcription is currently unknown and might be related to DNA methylation [29]. In fact, it has been reported that methylation regulates the expression of PD-1, PD-L1, and CTLA-4 in different cancers [30,31,32]. To date, The Cancer Genome Atlas (TCGA) cohort showed that TP53-associated immune prognostic signature is positively correlated with high expression of LAG-3 [33]. Additionally, in particular settings such as cutaneous T-cell lymphoma and melanoma, the LAG-3 expression is negatively regulated by microRNA-146 and histone deacetylase 6 inhibition [34,35].

## 3. Expression of LAG-3 on Immune Cells

The LAG-3 is expressed on Tregs cells, natural killer (NK), invariant NK T-cells, activated CD4^+^ T helper (Th) and cytotoxic CD8^+^ T lymphocytes, B cells, and plasmacytoid dendritic cells (pDCs) after stimulation by an antigen [13]. LAG-3 is not expressed on naïve T-cells such as PD-1 and CTLA-4. The rapid translocation of LAG-3 to the cell surface during T-cell activation appears to be facilitated by the significant intracellular storage of LAG-3 in addition to its association with the microtubule organizing center, early/recycling endosomes, and secretory lysosomes [36]. Moreover, LAG-3 is colocalized with CD4 in secretory lysosomes, microtubule organizing center, and recycling endosomes and appears faster than CD4 on the surface to suppress the function of T-cells when T-cells are activated [37]. This migration is mediated by the protein kinase C signaling pathway [38].

Annunziato and colleagues reported that LAG-3 is mainly expressed on activated CD4^+^ Th1, while Th0 and Th2 have weak or no LAG-3 expression [28]. Furthermore, this expression on activated CD4^+^ T-cells is found to be correlated with increased intracellular interferon-gamma (IFN-γ) production. What is more, LAG-3 expression is upregulated by cytokines such as IL-2, IL-7, and IL-12 which is found to be the strongest stimulus for its expression [27].

LAG-3 is overexpressed on tumor-infiltrating CD8^+^ T-cells in the TME of a wide range of solid tumors, such as ovarian cancer, renal cell carcinoma (RCC), hepatocellular carcinoma (HCC), melanoma, gastric cancer, follicular lymphoma (FL), non-small cell lung cancer (NSCLC), and head and neck squamous cell carcinoma (HNSCC) [39,40,41,42,43,44,45,46,47].

Studies have confirmed that the LAG-3 molecule potentiates the differentiation of Tregs [48]. This has been supported in studies in NSCLC patients, where they found high levels of LAG-3 expression in Tregs present in the TME as compared to those in the peripheral blood and unaffected organs. Furthermore, tumor-infiltrating Tregs expressing high levels of Foxp3^+^ secrete extreme amounts of immunosuppressive cytokines, thereby additionally suppressing anti-tumor activity [45,49].

The data on the relationship between LAG-3 and NK cells is scanty. However, this interaction was first described in mice where knockout of the LAG-3 gene generated decreased natural killer cells’ activity [50]. This was not replicated in human studies; besides, in another study, it was postulated that LAG-3 antibody actually did not have any impact on human natural killing [51]. On the contrary, LAG-3 has a more important influence on NKT cells. LAG-3 molecule induces signaling pathways that eventually downmodulate the activity of NKT cells by arresting the S phase in the cell cycle [52]. Other recent studies discovered that LAG-3 resulted in invariant NKT cell (iNKT) exhaustion as well as decreased IFN-γ production in patients having HIV [53].

Recent data showed that LAG-3 is uniformly expressed on pDCs, at levels higher than any subset of dendritic cells, almost 5-fold greater. LAG-3 mRNA was found in pDCs and not in lymphoid and myeloid DCs as well as in the red pulp of the spleen where pDCs are localized [10,54]. It acts as a negative regulator of pDC activation and expansion. This is through the interaction of LAG-3 on Treg cells with MHC-II, which is mediated by an ITAM suppressive signaling pathway, leading to inhibition of dendritic cell maturation [54]. Blackburn and colleagues reported that activated pDCs may secrete nearly five times more soluble LAG-3 than activated T-cells [55].

Moreover, LAG-3 is expressed on activated B lymphocytes and is produced endogenously. This expression depends on the activation of T-cells [56]. Lino and colleagues showed that LAG-3 is highly expressed in patients with chronic lymphocytic leukemia (CLL), a lymphoid neoplasm characterized by clonal expansion of mature B cells. LAG-3 is also found in natural regulatory plasma cells subset (LAG-3^+^CD138^hi^ plasma cells or Bregs) that differentiated in a B-cell receptor-dependent manner [57]. This subset of plasma cells (LAG-3^+^CD138^hi^) develops via an antigen-specific plasma cell epigenomic and transcriptional signature [57].

## 4. Expression of LAG-3 on Tumor Cells

The analysis of TCGA showed a wide expression range of LAG-3 in different cancer types. Moreover, high LAG-3 expression has been found in an extensive range of tumors including NSCLC, gastric cancer, colorectal cancer, breast cancer, ovarian cancer, HCC, RCC, FL, HNSCC, prostate cancer, pancreatic cancer, anal squamous cell carcinoma, and malignant pleural mesothelioma [40,41,42,43,44,45,58,59,60,61,62,63,64]. LAG-3 expression was mostly associated with poor clinicopathological associations and outcomes, including tumor progression, resistance, and metastasis.

Melanoma cells express MHC II molecules and attract tumor-specific CD4^+^ T-cells probably via LAG-3 interaction which negatively affects the response of CD8+ T-cells [65]. In a study by Camisachi et al., pDCs that express LAG-3 infiltrate the melanoma microenvironment and interact with HLA-DR-expressing tumor cells [66]. It was shown that LAG-3/MHC-II signaling in melanoma cells prevents their apoptosis by activating MAPK/ErK and PI3K/Akt survival pathways [39].

LAG-3 expression was also shown to be higher in glioma patients compared to healthy controls. In fact, LAG-3 is a member of the immunoglobulin superfamily of receptors that are present in microglia and neurons in the central nervous system [25]. It has been reported that LAG-3 expression was associated with elevated CD3^+^, CD8^+^, CD20^+^, and PD-1^+^ expression on TILs suggesting a correlation to an active inflammatory milieu [67]. Moreover, the high levels of LAG-3 expression were associated with lower OS and worse outcomes in both low-grade and high-grade gliomas [68]. Given its relatively high expression in gliomas and its role in promoting tumor growth, a study was conducted in a mouse glioblastoma model. It showed that LAG-3 inhibition is efficacious and can be used in combination with other ICIs [69,70]. Interestingly, in patients with glioblastoma multiforme, CD8A expression was associated with LAG-3 expression and not with PD-L1 expression in contrast with low-grade glioma [68].

In addition, patients with advanced HNSCC and high levels of soluble LAG3 were shown to have a poor prognosis independent of other factors [71]. Available data suggest that overexpression of LAG-3 on TILs is associated with high pathological grade, larger tumor size, and positive lymph nodes [61]. It was also shown that LAG-3 expression predominates on intratumoral Tregs that are CD4^+^CD25^hi^, found abundantly in the TME [72].

As for thyroid tumors, anaplastic thyroid cancers seem to have high LAG-3 expression which was directly correlated to PD-L1 expression, only in male patients. The overexpression of LAG-3 did not occur in the female patients’ group [73]. However, data on the significance of LAG-3 expression in papillary thyroid carcinomas are more discordant. One study showed that patients with papillary thyroid cancer have relatively lower expression of LAG-3 compared to normal healthy thyroid tissue [74]. Another study showed the opposite, where both anaplastic and papillary thyroid cancer showed increased LAG-3 expression, among other immune checkpoint mediators such as PD-L1, PD-L2, PD-1, and TIM-3 [75].

LAG-3 levels are significantly overexpressed in both small cell lung cancer (SCLC) as well as in NSCLC [76,77]. It was found that in NSCLC, intratumoral Tregs cells presented increased expression of inhibitory molecules including LAG-3, CTLA-4, and PD-1. Furthermore, NSCLC was associated with a higher number of CD4^+^CD25^+^FoxP3^+^ Tregs in the peripheral blood [77]. Another study showed that LAG-3 expressing CD4^+^CD25^−^ T-cells are present in the resected tumors and infiltrate metastatic sites more than the primary tumor [78]. Moreover, Ding and colleagues reported that LAG-3 upregulation was found in five of eight patients with NSCLC presenting acquired resistance to ICIs, suggesting that there might be a role for anti-LAG3 in that setting [79]. Moreover, in patients with lung adenocarcinoma, increased proportions of LAG-3^+^ cells are correlated with aggressive tumors, the presence of lymphovascular invasion, and nodal infiltration [41]. Moreover, one study evaluated the role and significance of PD-1, LAG-3, and TIM-3 expression in patients with NSCLC. As expected, the expression of these markers was lower in tumors with an EGFR mutation. Interestingly, in patients treated with PD-1 blockers, high LAG-3 expression was associated with shorter PFS [80]. In patients with squamous NSCLC, suppressed tumoral LAG3 expression was significantly associated with mutations in FA Complementation Group A (FANCA), Cut Like Homeobox 1 (CUX1), and NOTCH4 genes [81]. For patients with adenocarcinoma NSCLC, recurrence-free survival was worse in those with LAG3-positive TILs, compared to LAG3-negative [41].

In breast cancer, the expression of LAG-3 on TILS was reported in 11% of patients with breast cancer (BC) [42]. Burugu and colleagues also found that elevated LAG-3 expression is strongly associated with higher tumor grades, larger tumor size, and HER2^+^ and basal-like BC. In patients with triple-negative breast cancer (TNBC), LAG-3 is significantly upregulated and might be considered a biomarker [42]. However, its expression represents an independent favorable prognostic factor in patients with estrogen receptor-negative BC [42,82]. However, other studies do suggest that an immune phenotype with co-expression of PD-L1 and LAG-3 found in 15% of patients with TNBC infers a negative prognosis, more notably in patients with metastatic disease [83]. The frequency of co-expression is definitely not similar in all subtypes of BC; it seems to be the highest in patients with TNBC and the lowest in hormone receptors-positive BC [84].

In Hodgkin lymphoma (HL), a high level of LAG-3 on TILs and peripheral blood lymphocytes is associated with the suppression of EBV-specific T-cell-mediated immunity. CD4^+^ LAG-3 circulating T-cells were significantly higher in patients with HL presenting with active disease in comparison with those in remission [85]. One study demonstrated that LAG-3 and TIMs are almost always co-expressed in the TME of classical HL [86].

In FL, LAG-3 overexpression is associated with poor outcomes. Yang and colleagues demonstrated that LAG-3 was expressed on a subset of T-cells almost exclusively coming from to PD-1+ population. The expression of LAG-3 on CD4^+^ or CD8^+^ T-cells was substantially upregulated by the cytokine IL-12, leading to T-cell exhaustion. Interestingly, blocking PD-1 and LAG-3 enhanced the function of CD8+ T-cells, leading to increased IL-2 and IFN-γ production [60].

For gastric cancer, LAG-3 overexpression is actually associated with a favorable prognosis. Moreover, elevated sLAG-3 is positively linked to the secretion of IL-12 and IFN-γ and therefore inhibition of tumor growth. In addition, sLAG-3 proved to be a more valuable diagnostic marker than carcinoembryonic antigen (CEA) in this setting [87]. Nonetheless, LAG3 was described as a poor prognostic factor in patients with EBV-positive and MLH1 defective gastric cancer [88].

## 5. LAG-3 and Other Immune Checkpoints

Several studies have suggested that LAG-3 and PD-1 act synergistically to maintain immune homeostasis in both immune-mediated diseases and tumors [89,90]. They are both widely co-expressed on TILs, both CD4^+^ and CD8^+^ T-cells, in the TME [40,41,45]. The synergistic effect between LAG-3 and PD-1 has been described in murine melanoma, colorectal adenocarcinoma, and fibrosarcoma models where the dual blockade of both PD-1 and LAG-3 resulted in tumor eradication and prolonged survival in mice [91]. Moreover, in another study in a murine ovarian cancer model, dual antibody blockade or genetic knockout of LAG-3 and PD-1 resulted in enhanced CD8^+^ T-cell effector function and subsequent delayed tumor growth [92]. The co-expression of LAG-3 and PD-1 can result in exhausted and dysfunctional CD8^+^ T-cells; this was shown in NY-ESO-1 ovarian cancer samples where the synergy between LAG-3 and PD-1 generated dysfunctional CD8^+^ T-cells, reduced CD8^+^ T-cells activation, decreased cytokine release, and as a result an immune escape of tumor cells [43]. Another study looked at the relationship between LAG-3 and PD-1 expression; interestingly, over-expression of LAG-3 on TILs was associated with co-expression of PD-1/PD-L1. Moreover, patients with low expression of both LAG-3 and PD-L1 were shown to have a favorable prognosis [41]. In a nutshell, these preclinical data confirm the presence of synergy between LAG-3 and PD-1 and therefore serve as a basis for the development of combination treatment strategies in immune-oncology [93].

Another well-known cancer immune checkpoint, CTLA-4 also regulates anti-tumor immune responses to promote protective immunity and maintain tolerance, along with LAG-3. Both LAG-3 and CTLA-4 were shown to inhibit the TCR signaling pathway to eventually arrest cell cycle progression thereby negatively affecting T-cell homeostasis. They also induce the immunosuppressive activity of Tregs and have a substantial effect on DCs [94]. LAG-3 and CTLA-4 have a common intersection in their perspective signaling transduction pathways which may be the reason behind their functional similarity. In one study of anterior chamber-associated immune deviation (ACAID) mice models, LAG-3 and CTLA-4 were significantly upregulated on CD4^+^CD25^+^FOXp3^+^ Treg cells [95]. Moreover, induction of CD8^+^ Tregs by pDCs could result in coexpression of LAG-3 and CTLA-4, resulting in suppressed alloreactive T-cells through a CTLA-4-dependent mechanism [96]. Important data in graft versus host disease (GVHD), in humans and mice, demonstrated that dual blockade of CTLA-4 and LAG-3 using tetravalent Ig synergistically inhibited T-cell proliferation, suppressed T-cell responses thereby preventing the incidence of acute GVHD and decreasing GVHD mortality rates [97]. More importantly, a recent study assessing the therapeutic implication of ipilimumab, a CTLA-4 antibody, showed that ipilimumab increased the frequency of LAG-3 expression on tumor-infiltrating cells in metastatic melanoma patients [98].

## 6. Anti-LAG-3 Immunotherapy

Anti-LAG-3 directed therapies can be divided into three different categories: LAG-3-targeting monoclonal antibodies, bispecific LAG-3 antibodies, and LAG-3 fusion proteins. The majority of LAG-3-directed monoclonal antibodies are fully humanized IgG4 monoclonal antibodies with the exception of etigilimab, which is an IgG1 monoclonal antibodies [25]. It has been shown that LAG-3-targeting monoclonal antibodies suppress both IL-12 and IFN-γ production and that monoclonal antibodies inhibit the positive signal given via MHC-II to monocytes as well as the inhibition of T-cell response to IL-12 [99]. Available data suggest that anti-LAG-3 as monotherapy may not be an optimal treatment and combination therapy notably with PD-1 inhibitors is widely studied especially since LAG-3 and PD-1 are co-expressed on tumor-infiltrating CD8+ T-cells as previously mentioned. In fact, Blackburn and colleagues reported that the concomitant blockade of PD-1 and LAG-3 contributed to a significant increase in antigen-specific CD8+ T-cell numbers and function [55]. Relatlimab, a LAG-3-directed monoclonal antibody, is the first LAG-3 inhibitor approved in combination with nivolumab by the FDA for the treatment of previously untreated unresectable or metastatic melanoma based on the results of the RELATIVITY-047 trial [100]. On the other hand, bispecific antibodies achieved another milestone in the treatment of cancer patients consisting of two binding sites that target two different antigens or two different epitopes on the same antigen. To date, bispecific antibodies are mainly approved in hematologic malignancies such as teclistamab in multiple myeloma and glofitamab in diffuse large B-cell lymphoma [101,102]. Bispecific antibodies directed against PD-1 and LAG-3 are under evaluation in clinical practice. Regarding LAG-3 fusion proteins, eftilagimod alpha (IMP321) is a natural, soluble LAG-3 molecule with high affinity. It is the only soluble recombinant molecule clinically evaluated. It is an atypical ICI that targets antigen-presenting cells. IMP321 is a fusion molecule with LAG-3 extracellular domains combined with human immunoglobulin Fc region [103]. First, IMP321 vaccination has been described as a promising therapeutic strategy in inducing sustained immune responses by being a systemic APC activator to enhance DCs’ proliferation, lessen Tregs inhibitory activity, and allow for optimal cross antigen presenting to CD8^+^ T-cells as previously mentioned [104]. IMP321 recruits and activates effector innate and adaptive immune cells and stimulates T-cell proliferation leading to the production of IFN-γ, TNF-α, IL-1β, IL-6, CCL4, CCL5, and CCL2 [105]. Table 1 summarizes the major available data on LAG-3-directed therapies.

### 6.1. LAG-3 Fusion Proteins

Eftilagimod alpha (IMP321), is the only soluble LAG-3 protein that activated antigen-presenting cell (APC) that led to CD8 T-cell activation. The clinical efficacy of IMP321 as monotherapy was minimal. However, there is growing evidence that IMP321 is effective when combined with cytotoxic chemotherapy and vaccine-based strategies. It showed good tolerability and acceptable safety profiles when combined with gemcitabine for the treatment of patients with advanced pancreatic carcinoma [116]. In a phase 1 trial of patients with melanoma patients and who received melanoma antigen recognized by T-cell 1 (MART1) peptide vaccinations with or without IMP321, those treated with IMP321 presented decreased expression of PD-1, LAG-3, TIM-3, CD244, and CD160 [11]. Moreover, IMP321 was evaluated in combination with pembrolizumab in patients with metastatic melanoma. The authors reported that the combination was well tolerated and associated with encouraging anti-tumor activity [117]. The TACTI-002 study is a phase II trial evaluating the combination of eftilagimod alpha (IMP321) with pembrolizumab in patients with previously untreated unresectable or metastatic NSCLC, or recurrent PD-X refractory NSCLC or with recurrent or metastatic HNSCC. In a second-line setting in patients with PD-L1 unselected HNSCC, the combination was associated with an ORR of 39% (7/18 patients) with a good safety profile [106]. In the frontline setting for patients with NSCLC, the median duration of response (DOR) of 21.6 months and the median PFS was 6.6 months. The ORR increased with the PD-L1 tumor proportion score (TPS) [107]. Based on the results of the TACTI-002 trial, the FDA granted a fast-track designation to eftilagimod alpha in combination with pembrolizumab as a frontline treatment for patients with stage IIIB/IV NSCLC with a PD-L1 TPS score of at least 1%. In PD-1/PD-L1 refractory metastatic NSCLC, the combination was associated with encouraging PFS and OS. The median OS was 9.7 months, and the 6-month PFS rate was 25% [108].

### 6.2. Anti-LAG3 Monoclonal Antibodies

Relatlimab (BMS-986016) is a first-in-class humanized IgG4 antibody that binds to human LAG-3 with high affinity, inhibits its binding to MHCII, and restores the effector function of exhausted T-cells. It has been shown that relatlimab restores anti-leukemic responses mediated by T-cells and natural killer (NK), promotes leukemic cell depletion, and induces T-cell tumor necrosis factor (TNF)-α, IFN-γ and IL-2 cytokine [118]. In the RELATIVITY-020, a phase I/IIa trial, the combination of nivolumab and relatlimab was evaluated in patients with metastatic melanoma that had progressed on ICIs divided into two groups: patients had progressed on one line of ICI (D1) and patients on more than one PD-(L)1 containing regimens. The median duration of response was not reached in the D1 group and was 12.8 months in the D2 group. The 6-month PFS rate was 29.1% and 27.7%, respectively. This trial showed that nivolumab and relatlimab were associated with manageable safety profiles and demonstrated durable clinical activity in a subgroup of patients with heavily pretreated advanced melanoma who previously failed anti-PD-(L)1-containing regimens [109]. Then recently came RELATIVITY-047, a phase II/III, double-blinded, randomized study that evaluated relatlimab and nivolumab in untreated advanced melanoma. The study met its primary endpoint with an impressive median progression-free survival (mPFS) of 10.1 months with the combination group compared to only 4.6 months with nivolumab alone [100]. These results led to the first approval by the FDA of relatlimab in combination with nivolumab for the treatment of patients with untreated melanoma in March 2022. Amaria and colleagues reported the results of a prospective trial evaluating neoadjuvant nivolumab and relatlimab in patients with resectable melanoma. The combination was associated with a high pathologic complete response (pCR) rate. The 1-year and 2-year recurrence-free survival rates were 100% and 92%, respectively, for patients with any pathologic response, compared to 88% and 55% for those who did not achieve a pathologic response (*p* = 0.005) [110]. The NEOpredict-lung is a multicenter randomized phase II trial that compares preoperative nivolumab plus relatlimab versus nivolumab in patients with resectable NSCLC. The primary study endpoint was the feasibility of curatively intended surgery. Overall, 60 patients have been randomized. The trial showed that preoperative ICI with nivolumab and relatlimab is safe and feasible in patients with curatively resectable NSCLC [119]. The combination of nivolumab and relatlimab is now under clinical evaluation in multiple cancer types (Table 2).

Favezelimab (MK-4280) is a humanized, immunoglobulin G4, monoclonal antibody that inhibits the binding of LAG-3 to MHC class II. It leads to an increase in chemokine (CCL4, CXCL10, and CCL22) and cytokine (IFN-gamma, IL-2, IL-8, and TNF-alpha) production in T-cells [120]. In a first-in-human study, favezelimab in combination with pembrolizumab was associated with limited activity in patients with previously treated advanced microsatellite stable (MSS) colorectal cancer (CRC) [121]. The combination is also under investigation in patients with relapsed and/or refractory (R/R) hematological malignancies (NCT03598608). In PD-1-naïve R/R classical HL, the combination of favezelimab and pembrolizumab was associated with an ORR of 73% (22/30 patients) including 23% of CR at a median follow-up of 13.5 months. The median PFS was 19 months, and the median OS was not reached. The 12-month OS rate was 96% [111]. This efficacy was also shown in the cohort of patients with R/R classical HL who failed an anti-PD-1 treatment suggesting the combination may reinduce a response in these patients. The ORR was 31% (9/29) including 7% of CR after a median follow-up of 16.5 months. The median PFS and OS were 9 months and 26 months, respectively [112].

Fianlimab (REGN3767) is a fully human IgG4, hinge-stabilized, high-affinity, monoclonal antibody that targets LAG-3. The combination of fianlimab with cemiplimab, a PD-1 inhibitor, was evaluated in a phase I trial of patients with advanced melanoma. The combination was associated with an ORR of 63.6% (21/33) in PD-(L)-1 naïve patients and 13.3% in patients previously treated with a PD-(L)-1 inhibitor. The median PFS and duration of response of the PD-(L)-1 naïve had not been reached. The combination was well tolerated and associated with a good safety profile [113].

Ieramilimab (LAG525) is another humanized IgG4 monoclonal antibody that binds to LAG-3. It has been evaluated with or without spartalizumab (PDR001), a PD-1 inhibitor, in patients with advanced malignancies. LAG525 has a good safety profile as monotherapy or in combination with PDR001. However, it was associated with modest clinical activity with an ORR of 10% in the combination arm [122].

Other monoclonal antibodies targeting LAG-3 are also under clinical investigation such HLX26 (NCT05078593 and NCT05400265), IBI110 in diffuse large B cell lymphoma (NCT05039658), INCAGN02385 (NCT03538028, NCT04370704, NCT05287113, NCT04586244), Sym022 (NCT03489369, NCT03311412, NCT04641871), TSR-033 (NCT03250832, NCT02817633).

### 6.3. Anti-LAG3 Bispecific Antibodies

Tebotelimab (MGD013) is a bispecific antibody targeting LAG-3 and PD-1 with high affinity and prolonged half-life. It is under clinical development in patients with unresectable neoplasm. It has been demonstrated that treatment with tebotelimab resulted in a significant increase in the serum levels of IFN-γ, expansion of circulating CD3^+^CD8^+^ and CD3^+^CD4^−^CD8^−^ T-cell subpopulations as well as cytolytic markers such as grazyme B and perforin [123]. It has been evaluated in a phase 1/2 dose escalation and expansion trial in patients with advanced HCC who failed prior targeted therapy and/or immunotherapy. In the ICI-naïve cohort, the ORR was 13.3% (4/30 patients), and the median PFS of 3.1 months. However, in the ICI-experienced cohort, the ORR was 3.3%, and the median (1/30 patients). The treatment was associated with a manageable safety profile [114]. Moreover, tebotelimab in combination with margetuximab, an investigational anti-HER2 monoclonal antibody showed encouraging clinical activity in patients with advanced HER2+ neoplasms. The ORR was 40% (8/20 patients) [124]. Multiple ongoing phase I or II trials are investigating tebotelimab in patients with melanoma (NCT04653038), liver cancer (NCT04212221), head and neck (NCT04634825, NCT04082364), as monotherapy or in combination with niraparib (selective PARP1/2 inhibitor), or brivanib alaninate (multitargeted tyrosine kinase inhibitor), or enoblituzumab (anti-B7-H3 antibody) (Table 3).

RO7247669 (NP41300) is a novel bispecific antibody that delivers dual checkpoint inhibition through monovalent high affinity binding to PD-1, and monovalent binding to LAG-3 allowing a unique avidity-mediated selectivity gain. In a phase I trial in patients with advanced and/or metastatic solid tumors (NCT04140500). Thirty-five patients were treated of whom 40% received at least three prior lines of treatment and 34.3% received prior ICI treatment. The ORR was 17.1% of patients (6/35) and the disease control rate (DCR) was 51.4%. Treatment-related grade 3 AEs occurred in six patients (17.1%) and no grade 4–5 AEs were reported. No dose-limiting toxicity has been observed [115].

Pavunalimab (XmAb22841) is a novel LAG-3/CTLA-4 bispecific antibody that is under investigation in a phase I trial (DUET-4) as monotherapy or in combination with pembrolizumab in patients with selected advanced solid tumors (NCT03849469). The combination of pavunalimab and XmAb23104 (PD-1/ICOS) antibody is under evaluation in a phase 1/2 trial in patients with melanoma who previously received prior ICIs (NCT05695898) (Table 3).

FS118 is a LAG-3/PD-1 bispecific antibody and was developed as a single agent in a phase I/II trial in patients with advanced malignancies (NCT03440437).

EMB-02 is another PD-1/LAG-3 bispecific antibody and is currently under clinical development in a phase I/II trial in advanced solid tumors (NCT04618393).

## 7. Conclusions

LAG-3 is now considered a promising therapeutic target with two drugs that had been approved by the FDA in combination with PD-1 inhibitors for the treatment of patients with solid tumors. Several clinical trials are ongoing and are evaluating different combinations in oncology and hematology. Interestingly, The FDA approved relatlimab and nivolumab in patients with previously untreated advanced melanoma in March 2002 which is the second combination approved in this setting after nivolumab plus ipilimumab, an anti-CTLA-4 monoclonal antibody. To date, there is no direct comparison between the two combinations. The combination of three ICIs targeting PD-1/PD-L1, CTLA-4, and LAG-3 may be an option for oncologic patients. An ongoing phase II trial is evaluating the efficacy and the safety of the combination of ipilimumab, nivolumab, and relatlimab in unresectable stage III or stage IV melanoma (NCT05428007).

Despite the surge of research involving targeting LAG-3, there still remain several important unanswered questions.

First, there are still missing gaps in the mechanisms by which LAG-3 mediates the TCR signaling pathway and function. Given the unique intracellular cytoplasmic domain of LAG-3, the KEEILE motif, and its important role, a thorough understanding of the biology of LAG-3 is crucial to moving forward in its implications clinically.

Second, the efficacy demonstrated in the RELATIVITY-047 trial raises the question of the possibility of novel biomarker testing. It is still unclear whether LAG-3 positivity will be useful in informing therapeutic choices. Research is still needed to deepen our understanding of LAG-3 expression and its clinical implications. It remains to be seen whether LAG-3 testing can serve as a robust predictive biomarker to predict response.

## Figures and Tables

**Table 1:** Summarizes major trials and available data concerning the efficacy of LAG-3-directed therapies (eftilagimod alpha, relatlimab, favezelimab, and fianlimab).

Drug	Class	Treatment	Phase	Population	No of Pts	Outcome	AEs
Eftilagimod alpha (IMP321) [106]	LAG-3 fusion protein	Eftilagimod alpha + pembrolizumab	II	2nd line PD-L1 unselected HNSCC	18	ORR: 39%	Cough (29%) Asthenia (24%) Dyspnea (18%)
Eftilagimod alpha (IMP321) [107]	LAG-3 fusion protein	Eftilagimod alpha + pembrolizumab	II	1st line metastatic NSCLC	110	ORR (ITT): 40.4% ORR (TPS < 1%): 31% ORR (TPS 1–49%): 45% ORR (TPS > 50%): 55%	Pruritus (20%) Asthenia (19%) Rash (13%) Diarrhea (11%)
Eft-ilagimod alpha (IMP321) [108]	LAG-3 fusion protein	Eftilagimod alpha + pembrolizumab	II	2nd line NSCLC refractor to PD1/PD-L1 inhibitors	36	Median OS: 9.7 months	
Relatlimab (RELATIVITY-020) [109]	LAG-3 monoclonal antibody	Relatlimab + nivolumab	I/II	Metastatic melanoma progressing after PD-1/PD-L1 inhibitors	582 D1:354 D2:164	ORR: D1: 12% D2: 9%	Grade 3 or more: D1: 15% D2: 13%
Relatlimab (RELATIVITY-047) [100]	LAG-3 monoclonal antibody	Relatlimab + nivolumab vs. nivolumab	II/III	Untreated advanced melanoma	714	mPFS: 10.1 vs. 4.6 months	Grade 3 or more: 19% in combination vs. 10% in monotherapy
Relatlimab [110]	LAG-3 monoclonal antibody	Relatlimab + nivolumab	II	Neoadjuvant treatment in operable melanoma	60	pCR: 57% overall PR: 70%	No grade 3 AEs
Favezelimab [111]	LAG-3 monoclonal antibody	Favezelimab + pembrolizumab	I/II	PD-1 inhibitor naïve R/R cHL	30	ORR: 73% mPFS: 19 months	Grade 3: 20%
Favezelimab [112]	LAG-3 monoclonal antibody	Favezelimab + pembrolizumab	I/II	Prior PD-1 inhibitor R/R cHL	29	ORR: 31% mPFS: 9 months	Grade 3: 18%
Fianlimab [113]	LAG-3 monoclonal antibody	Fianlimab + cemiplimab	I	Advanced melanoma	33	ORR: 64% in PD-1/PD-L1i-naïve ORR: 13% in PD-1/PD-L1i-resistant	Grade 3 or more: 35%
Tebotelimab [114]	LAG-3/PD-1 bispecific antibody	Tebotelimab	I/II	Advanced HCC	63	ORR: 13% in ICI-naïve 3% in ICI-refractory	Grade 3 or more: 19%
RO7247669 [115]	LAG-3/PD-1 bispecific antibody	RO7247669	I	Advanced and/or metastatic solid tumors	35	ORR: 17%	Grade 3 or more: 17%

AE: adverse events; No: number; pts: patients; D1: one line of ICI; D2: more than one line of PD-1/PD-L1 inhibitors; pCR: pathologic complete response; PR: pathologic response; R/R: relapsed and/or refractory; cHL: classical Hodgkin lymphoma; HCC: hepatocellular carcinoma; PD-1: programmed death-1; PD-L1: programmed death ligand-1; NSCLC: non-small cell lung cancer; ORR: overall response rate; TPS: tumor proportion score; ITT: intention-to-treat; HNSCC: head and neck squamous cell carcinoma; OS: overall survival; mPFS: median progression-free survival; ICI: immune checkpoint inhibitors.

**Table 2:** Resumes major ongoing trials evaluating the combination of nivolumab with relatlimab in several types of cancers.

Reference	Drugs	Phase	N	Population	Primary Endpoint
NCT05002569 (RELATIVITY-098)	Nivolumab + relatlimab vs. nivolumab	3	1050	Adjuvant therapy after resection of stage III-IV melanoma	RFS
NCT05625399 (RELATIVITY-127)	Sc nivolumab + relatlimab vs. IV nivolumab + relatlimab	3	814	Previously untreated metastatic or unresectable melanoma	ORR
NCT05328908 (RELATIVITY-123)	Nivolumab + relatlimab vs. regorafenib or TAS-102	3	700	Later-lines of metastatic CRC	OS in PD-L1 > 1 OS
NCT05337137 (RELATIVITY-106)	Nivolumab + relatlimab + bevacizumab	1/2	162	Treatment-naïve advanced or metastatic HCC	DLT PFS
NCT04205552 (NEOpredict)	Nivolumab alone or + relatlimab	2	90	Neoadjuvant in resectable NSCLC	Feasibility
NCT05704647	Nivolumab + relatlimab	2	30	Melanoma with active brain metastases	AEs
NCT05148546 (NESCIO)	Nivolumab alone or + ipilimumab or relatlimab	2	69	Neoadjuvant in clear cell RCC at risk for recurrence or distant metastases	PRR
NCT03610711 (REACTION)	Nivolumab alone or + relatlimab	1/2	21	Advanced esophagogastric cancer	Change in infiltrating CD8+ T-cell density
NCT04552223	Nivolumab + relatlimab	2	27	Metastatic uveal melanoma	ORR
NCT05418972 (Neo ReNi II)	Nivolumab + relatlimab	2	20	Neoadjuvant, high-risk, stage II melanoma	PRR
NCT03743766	Nivolumab + relatlimab	2	42	Metastatic melanoma naïve to prior immunotherapy	ORR
NCT03607890	Nivolumab + relatlimab	2	42	Advanced mismatch repair deficient cancers resistant to prior PD-(L)1 inhibitor	ORR
NCT04658147	Nivolumab with or without relatlimab	1	20	Perioperative potentially resectable HCC	% of patients who complete pre-op treatment
NCT04095208 (CONGRATS)	Nivolumab + relatlimab	2	67	Advanced or metastatic soft-tissue sarcoma	ORR, DCR
NCT04913922 (AARON)	Nivolumab + relatlimab + 5-Azacytidine	2	30	R/R AML, untreated older AML patients	MTD, DLT, ORR
NCT05176483 (STELLAR-002)	XL092 + nivolumab + ipilimumab or relatlimab	1	1078	Unresectable advanced or metastatic solid tumors	AEs ORR PFS OS
NCT03623854	Nivolumab + relatlimab	2	20	Advanced chordoma	ORR
NCT05255601 (RELATIVITY-069)	Nivolumab + relatlimab	1/2	68	pediatric and young adult with R/R cHL and NHL	DLT MTD
NCT05704933	Nivolumab + ipilimumab or relatlimab	1	24	Surgically resectable melanoma brain metastases	Feasibility comparison of immune cell population
NCT05428007	Sarilumab + ipilimumab + nivolumab + relatlimab	2	69	Unresectable stage III or stage IV melanoma	irAE ORR
NCT04204837	Nivolumab alone or + relatlimab	2	61	LA or metastatic SCC of the skin	ORR
NCT03521830	Nivolumab alone or + relatlimab or ipilimumab	2	40	LA or metastatic basal cell carcinoma	ORR
NCT03642067	Nivolumab + relatlimab	2	96	MSS advanced CRC	ORR
NCT03026140 (NICHE)	Nivolumab + ipilimumab or relatlimab	2	268	Neoadjuvant combination in early stage CRC	AEs DFS
NCT04623775	Nivolumab + relatlimab + chemotherapy vs. nivolumab + chemotherapy	2	420	First-line in stage IV or recurrent NSCLC	TRAE ORR
NCT04080804	Nivolumab alone or in combination with relatlimab or ipilimumab	2	60	Neoadjuvant in LA resectable HNSCC	AEs

ORR: objective response rate; AEs: adverse events; DLTs: dose-limiting toxicities; DCR: disease control rate; DOR: duration of response; PFS: progression-free survival; OS: overall survival; TEAEs: treatment-emergent adverse events; irAEs: immune-related adverse events; PRR: pathological response rate; MTD: maximal tolerated dose; RFS: recurrence-free survival; MSS: microsatellite stable; CRC: colorectal cancer; TRAE: treatment-related adverse events; DFS: disease-free survival; LA: locally advanced; SCC: squamous cell carcinoma; NSCLC: non-small cell lung cancer; HNSCC: head and neck squamous cell carcinoma; cHL: classical Hodgkin Lymphoma; NHL: non-Hodgkin lymphoma; PRR: pathological response rate; RCC: renal cell carcinoma; HCC: hepatocellular carcinoma.

**Table 3:** Summarizes major ongoing trials evaluating LAG-3-directed therapies except the combination of nivolumab and relatlimab.

Reference	Drugs	Phase	N	Population	Primary Endpoint
NCT04811027 (TACTI-003)	Eftilagimod alpha + pembrolizumab	2	154	First-line: unresectable R/M HNSCC	ORR
NCT04252768 (AIPAC-002)	Eftilagimod alpha + paclitaxel	1	24	HR+ metastatic breast cancer	Safety and tolerability
NCT05747794 (AIPAC-003)	Eftilagimod alpha or placebo + paclitaxel	3	849	HER2-neg/low metastatic breast cancer	OS, Aes, OBD
NCT03252938	Eftilagimod alpha	1	45	IT, IP, SC alone or in combination in advanced solid tumors	Feasibility rate
NCT03005782	Fianlimab with or without REGN2810 (Anti-PD1)	1	333	Advanced malignancies	DLTs AEs Serious AEs
NCT05352672	Fianlimab + cemiplimab vs. pembrolizumab	3	1590	Previously untreated unresectable LA or metastatic melanoma	PFS
NCT04140500	RO7247669 (PD1-LAG3 bispecific antibody)	1	320	Advanced and/or metastatic solid tumors	DLTs, AEs, ORR, DCR, DOR, PFS
NCT05419388	RO7247669	1/2	80	Previously untreated unresectable or metastatic melanoma	PFS
NCT05645692	RO7247669 +/− tiragolumab vs. atezolizumab	2	240	Previously untreated advanced or metastatic UC ineligible for platinum-containing chemotherapy	ORR
NCT04785820	RO7247669 vs. RO7121661 (PD1-TIM3 bispecific antibody) vs. nivolumab	2	210	Relapsed or intolerant to platinum-containing regimens in A/M SCCE	OS
NCT05508867 (KEYFORM-008)	favezelimab + pembrolizumab vs. physician’s choice chemotherapy	3	360	PD-(L)1-refractory, R/R classical Hodgkin lymphoma	PFS
NCT05064059 (MK-4280A-007)	favezelimab + pembrolizumab vs. SOC	3	432	Previously treated metastatic PD-L1 positive CRC	OS
NCT03598608 (MK-4280-003)	Favezelimab + pembrolizumab	1/2	174	Hematologic malignancies	DLTs, AEs, treatment discontinuation due to AEs
NCT04938817 (MK-3475-B98/KEYNOTE-B98)	Pembrolizumab + favezelimab or quavonlimab	1/2	80	PD-(L)1 refractory extensive-stage SCLC	DLTs AEs TRAEs ORR
NCT05695898	XmAb23104 (PD1-ICOS) + XmAb22841 (CTLA-4-LAG3)	1/2	46	Metastatic melanoma refractory to prior ICI with and without CNS disease	TEAEs, irAEs, DLTs
NCT04150965	BMS-986016 + Pomalidomide + dexamethasone (Arm B)	1/2	104	Relapsed and/or refractory MM	ORR, AEs

ORR: objective response rate; AEs: adverse events; DLTs: dose-limiting toxicities; DCR: disease control rate; DOR: duration of response; PFS: progression-free survival; IT: intra-tumoral; IP: intra-peritoneal; SC: subcutaneous; OBD: optimal biological dose; TEAEs: treatment-emergent adverse events; TRAEs: treatment-related adverse events; irAE: immune-related adverse event; CNS: central nervous system; SCLC: small cell lung cancer; MM: multiple myeloma; ICI: immune checkpoint inhibitor; CRC: colorectal cancer; OS: overall survival; R/R: relapsed and/or refractory; SCCE: squamous cell carcinoma of the esophagus.

## Data Availability

Not applicable.
